# Outcomes of a Self-Assessment Intervention Approach Based on the Scale for Achievement Motive in Rehabilitation and Interview Methods in Patients After Total Hip Arthroplasty: A Retrospective Case Series

**DOI:** 10.7759/cureus.96143

**Published:** 2025-11-05

**Authors:** Nobuyuki Sano, Kentaro Matsumoto

**Affiliations:** 1 Department of Occupational Therapy, Faculty of Medical Science, Fukuoka International University of Health and Welfare, Fukuoka, JPN; 2 Rehabilitation Department, Takagi Hospital, Okawa, JPN

**Keywords:** achievement motivation, behavioral modification, intervention strategy, semi-structured interview method, total hip arthroplasty

## Abstract

Introduction

In occupational therapy that supports client autonomy, assessment and intervention regarding their psychological state are important. The Scale for Achievement Motive in Rehabilitation (SAMR) is used as an assessment tool for one’s motivation to pursue goals. By combining self-assessment with SAMR and a semi-structured interview method, intervention strategies have been developed that can enhance the client's motivation toward their goals and participation in daily activities. Our aim was to compare the changes in achievement motive and activities of daily living before and after this intervention method.

Methods

This is a retrospective case series involving 11 patients (all female, average age 62.27±5.02 years) who had undergone total hip arthroplasty. An occupational therapy intervention combining SAMR and interview methods was conducted for 6 to 10 weeks, and changes in achievement motivation and activities of daily living before and after the intervention were compared. The measurement used SAMR and the Functional Independence Measure (FIM). Information such as length of hospital stay and mobility methods at admission and discharge was collected. Paired t-tests were conducted for the initial and follow-up SAMR scores, as well as for the FIM at the start of intervention and at discharge. Furthermore, Pearson’s correlation analysis was used to examine the relationship between the initial SAMR total score and the change in FIM score.

Results

There was a significant improvement in the total SAMR scores in all cases (effect size: Cohen's d = 1.29, 95% confidence intervals: 0.27-2.30). Additionally, while the correlation coefficient between the initial SAMR total score and the changes in the FIM was not statistically significant (p = 0.14), the correlation coefficient was r = 0.476, which represents a medium effect size.

Conclusions

This report highlights the importance of providing support that aligns with the patient’s current level of achievement motive through interviews, allowing patients to consider goal setting and action planning for themselves.

## Introduction

Rehabilitation is defined in the Convention on the Rights of Persons with Disabilities as “take effective and appropriate measures, including through peer support, to enable persons with disabilities to attain and maintain maximum independence, full physical, mental, social and vocational ability, and full inclusion and participation in all aspects of life” [1]. In rehabilitation support, person-centered therapy involves assessing and addressing each individual’s unique experiences, goals, and values in order to enable independence and self-determination [2]. Based on these principles, occupational therapists, as rehabilitation professionals, are expected to provide support that enables individuals to build meaningful daily life activities. To demonstrate the effectiveness of occupational therapy focusing on supporting the independence and self-determination of individuals, it is necessary to evaluate psychological states such as motivation and drive, to intervene appropriately, and to examine what effects these interventions have [3].

Among the motivation and drive of individuals, the desire to strive toward achieving goals is called "achievement motivation" in the field of psychology. This was defined by Murray as the capacity “to overcome obstacles, to exercise power, to strive to do something difficult as well as quickly as possible" [4]. In the field of rehabilitation as well, enhancing achievement motive has been introduced as a role of counseling, and the importance of assisting with goal setting and self-directed action selection has been advocated [5,6]. As a method for assessing the achievement motivation of individuals receiving rehabilitation support, a self-administered questionnaire called the Scale for Achievement Motive in Rehabilitation (SAMR) has been developed [7,8]. The SAMR evaluates the state of achievement motivation on six levels based on the total score (ranging from 10 to 70 points) and interprets the characteristics of achievement motivation according to two factors (self-mastery-derived and means/process-oriented-derived). Self-mastery-derived factor refers to making an effort to enhance one’s own abilities and intelligence; the means/process-oriented-derived factor emphasizes the willingness to follow a rehabilitation program to achieve one’s goals [9]. Cases using the SAMR have been reported in which the characteristics and intensity of motivation are interpreted from the total and factor scores, and interventions are implemented to share goals and actions that draw their motivation [10,11]. Furthermore, by using the results of the self-assessment with SAMR and combining it with a semi-structured interview method that sets goals, action plans, and support from those around the person to promote occupational participation, an intervention procedure has been developed that can increase the motivation toward goals and participation in daily activities for individuals [11]. Activities of daily living (ADL) are fundamental to leading a social life, essential for basic survival and well-being, and are expected to improve through occupational therapy interventions [3]. However, as far as we know, reports on interventions using SAMR have only been conducted as single-case studies. Therefore, there is a possibility that the effects of interventions using SAMR may be limited to specific cases only.

In this study, we conducted a retrospective case series at a single hospital involving multiple patients who had undergone total hip arthroplasty (THA), combining the use of SAMR and interview methods. By comparing changes in achievement motivation and behavioral modification, we examined the characteristics of this practical approach.

## Materials and methods

Participants

This study adopted a purposive sampling technique, in which patients who met the inclusion criteria and received occupational therapy after THA were included. The inclusion criteria were patients who underwent THA at Yanagawa Rehabilitation Hospital between September 2019 and July 2020, received preoperative activities of daily living (ADL) guidance for postoperative care from occupational therapists, and started occupational therapy on the day after surgery. The exclusion criteria were individuals with significant cognitive impairment, defined as a score of less than 24 on the Mini-Mental State Examination-Japanese (MMSE-J) [12,13], administered at the start of occupational therapy following THA. THA is a surgical procedure that replaces both the worn or damaged acetabulum and femoral head with artificial joint implants, primarily aimed at relieving pain and restoring function in patients with end-stage hip disorders such as osteoarthritis. The reason for selecting patients who had undergone THA as subjects in this study was that, at this hospital, the patients receiving THA have a limited range of ages and diagnoses, and postoperative treatment interventions are carried out in accordance with the hospital’s standardized clinical pathways, which was thought to minimize individual differences in the course of intervention.

Data collection and measurements

Achievement motivation was assessed using SAMR. SAMR is a self-assessment tool with 10 questions, rated on a 7-point scale from "strongly agree (7 points)" to "strongly disagree (1 point)." It allows calculation of a total score (range: 10-70) as well as scores for the self-mastery-derived factor (items 1-6) and the means/process-oriented-derived factor (items 7-10) [7]. Furthermore, based on the standardized total score of the SAMR, the level of achievement motivation can be classified into six levels: 66 points or higher is considered "high level," 57 to 65 points is "somewhat high," 48 to 56 points is "average," 40 to 47 points is "somewhat low," 31 to 39 points is "low," and 30 points or lower is interpreted as a "very low" level of achievement motivation [14]. The SAMR was originally developed by the authors and is available for academic and clinical use without restriction.

For the evaluation of ADL, the Functional Independence Measure (FIM) was used, and scores at the start of intervention and at discharge were collected. FIM was used to assess the patients’ level of independence in ADL. The FIM consists of 18 items, including 13 motor and 5 cognition domains. Each item is scored on a 7-point ordinal scale ranging from 1 (total assistance) to 7 (complete independence), with total scores ranging from 18 to 126 points. Higher scores indicate greater functional independence [15,16].

In addition, the number of days from surgery to the start of occupational therapy, the length of hospital stays (days), and the means of mobility at admission and discharge were collected from medical records. Furthermore, changes in goals leading to occupational participation and the action plans devised by the participants themselves at the time of the initial and subsequent evaluations were confirmed.

Intervention process

**Figure 1:**
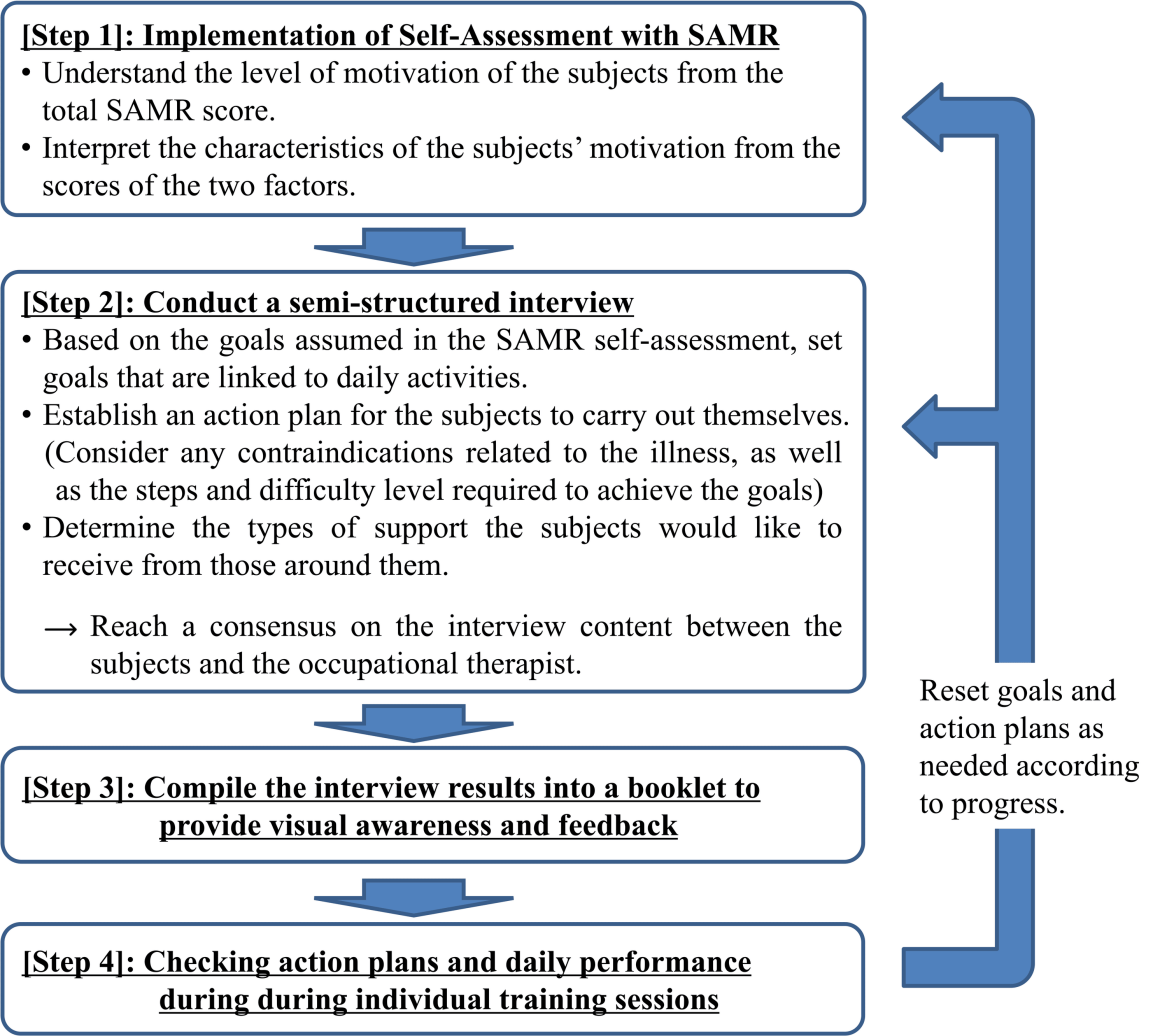
Flow of intervention using self-assessment of the SAMR and semi-structured interview method

Occupational therapy was initiated for all cases starting the day after surgery, conducted once daily, seven days a week, with each session lasting approximately 40 to 60 minutes. After observing treatment progress in the general ward for the first week postoperatively, patients were transferred to the convalescent rehabilitation ward starting the following week. Interventions using SAMR and the interview method were conducted based on the explicitly defined intervention process [11] (Figure 1). In this report, after patients had passed the acute phase following THA and were prescribed occupational therapy, they were asked to complete a self-assessment using SAMR within approximately 3-5 days. After that, using an interview method during the regular occupational therapy session for about 20 minutes, the occupational therapist and the patients discussed and set specific goals to connect with activities that are meaningful to the patients, based on the goals envisioned during the SAMR assessment. They also discussed and established an action plan outlining the necessary steps to achieve these goals, as well as the types of support the patients would like to receive from those around them. To reinforce awareness visually, a booklet summarizing the interview results was distributed to each patient for their confirmation. In each occupational therapy session, activities related to the goals were carried out for more than half of the session time. The follow-up SAMR self-assessment and interview were conducted approximately 30-40 days later. This intervention was carried out entirely by the occupational therapist (KM), who is a co-author.

Statistical analysis

For analysis, the Jarque-Bera test was used to confirm the normality of the data. Paired t-tests were conducted for the initial and follow-up SAMR scores (total score, self-mastery-derived factor, and means/process-oriented-derived factor), as well as for the FIM total score at the start of intervention and at discharge (with a significance level of less than 5%). Effect size changes (Cohen's d) were calculated with 95% confidence intervals (CI) to represent the magnitude of change before and after the intervention within the same participants. Furthermore, the amount of change in total FIM score from the start of intervention to discharge was calculated, and Pearson’s correlation analysis was used to examine the relationship between the initial SAMR total score and the change in FIM score. For confirming effect sizes, Cohen's d was assessed as "small" for values of 0.2 or higher, "medium" for 0.5 or higher, and "large" for 0.8 or higher [17]. Regarding the correlation coefficient r, values of 0.1 or higher were considered "small," 0.3 or higher "medium," and 0.5 or higher "large" [17]. All analyses were conducted using HAD17 or R.

Ethical considerations

This study was approved by the Institutional Review Board of Fukuoka International University of Health and Welfare (Approval No. 25-TG-007). In accordance with the Ethical Guidelines for Medical and Health Research Involving Human Subjects in Japan and the Declaration of Helsinki, informed consent was obtained through an opt-out process. Information regarding the study was posted on the hospital bulletin board and the hospital’s official website, allowing patients to decline participation.

## Results

Ultimately, 11 patients were selected (all female, with an average age of 62.27±5.02 years), all of whom were diagnosed with osteoarthritis of the hip (with four cases on the right side and seven on the left side) (Table 1). The average length of hospital stay for all cases was 62.82±8.93 days.

**Table 1:** Participant characteristics MMSE-J: Mini Mental State Examination-Japanese

	Case 1	Case 2	Case 3	Case 4	Case 5	Case 6	Case 7	Case 8	Case 9	Case 10	Case 11
Gender	Female	Female	Female	Female	Female	Female	Female	Female	Female	Female	Female
Age	54	69	58	65	64	61	67	66	64	63	54
Surgical side	Left	Right	Left	Left	Right	Left	Left	Left	Right	Right	Left
Length of hospital stay	63	72	65	62	68	75	66	50	58	67	45
MMSE-J	30	29	30	30	30	29	29	30	29	30	30
Means of mobility at admission	With a cane	With a cane	Independent	Independent	Wheelchair	Wheelchair	With a cane	Independent	Independent	Independent	Independent
Means of mobility at discharge	With a cane	With a cane	With a cane	With a cane	With a cane	Wheelchair and/or With a cane	With a cane	With a cane	With a cane	With a cane	With a cane
Number of days from surgery to this intervention	3	4	3	3	3	3	5	4	3	4	5

For the patients in this study, most of the goals identified through the intervention were related to household activities (e.g., cooking, laundry) and returning to work. The action plans involved setting specific scenarios and frequencies within the ward that aligned with these goals and focused on improving physical functions. For the types of support from those around the patient, professionals (e.g., therapists, nurses) and family members were assigned to observe and encourage independent training.

**Figure 2:**
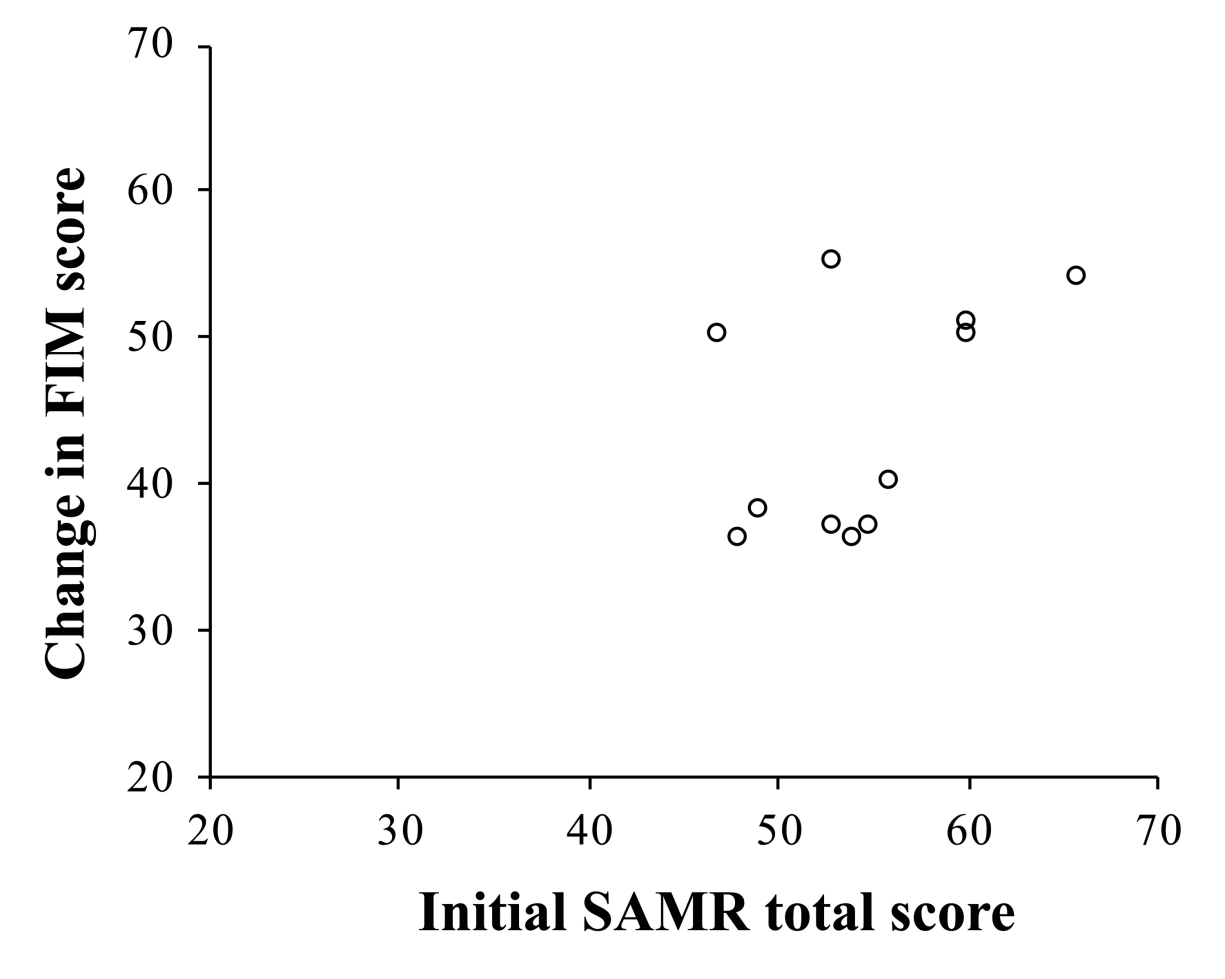
The correlation coefficient with the initial total SAMR score and the amount of change in FIM Pearson’s correlation coefficient: r = 0.476, p = 0.14.

Normality was confirmed for all variables. In SAMR, the initial total score was 54.64±5.73 points, with the self-mastery-derived factor at 31.45±3.24 points and the means/process-oriented-derived factor at 23.18±2.93 points (Table 2). The follow-up total score was 61.73±5.37 points (effect size: Cohen's d = 1.29, 95% CI: 0.27-2.30), the self-mastery-derived factor was 36.45±2.91 points (effect size: Cohen's d = 1.64, 95% CI: 0.57-2.71), and the means/process-oriented-derived factor was 25.27±3.35 points (effect size: Cohen's d = 0.67, 95% CI: -0.26-1.61). “Large” effects were observed for the total score and the self-mastery-derived factor, and a “medium” effect was observed for the means/process-oriented-derived factor. Regarding the FIM, individuals with cognitive impairment were excluded based on the exclusion criteria, and since all patients in this study scored full marks on the cognition subscale both before and after the intervention, only the total FIM score was analyzed. The total FIM score at the start of intervention was 75.36±8.74 points, and at discharge it was 119.36±1.43 points (effect size: Cohen's d = 7.09, 95% CI: 4.45-9.72), indicating a “large” effect when comparing before and after intervention. The amount of change in FIM was 44.00±7.87 points, and the correlation coefficient with the initial total SAMR score was r = 0.476, which was judged to be “medium” in effect size, but statistical significance was not observed (p = 0.14) (Figure 2).

**Table 2:** Changes in SAMR and FIM scores of participants SAMR: Scale for Achievement Motive in Rehabilitation,  FIM: Functional Independence Measure, Self-mastery: Self-mastery-derived factor, Means/process: Means/process-oriented-derived factor, SD: Standard deviation, 95%CI: 95% confidence intervals. The effect size was calculated using Cohen's d.

	SAMR total score	Self-mastery score	Means/process score	FIM total score
	Initial/Follow-up/Amount of change	Initial/Follow-up/Amount of change	Initial/Follow-up/Amount of change	At start of intervention/ At discharge/Amount of change
Case 1	47/60/13	27/35/8	20/25/5	69/119/50
Case 2	60/68/8	34/41/7	26/27/1	67/118/51
Case 3	56/63/7	33/38/5	23/25/2	81/121/40
Case 4	55/64/9	30/36/6	25/28/3	83/120/37
Case 5	54/66/12	30/38/8	24/28/4	82/118/36
Case 6	60/66/6	35/39/4	25/27/2	67/117/50
Case 7	66/69/3	38/39/1	28/30/2	66/120/54
Case 8	49/55/6	31/36/5	18/19/1	83/121/38
Case 9	53/55/2	30/31/1	23/24/1	83/120/37
Case 10	53/58/5	30/33/3	23/25/2	63/118/55
Case 11	48/55/7	28/35/7	20/20/0	85/121/36
Average	54.64/61.73/7.09	31.45/36.45/5.00	23.18/25.27/2.09	75.36/119.36/44.00
SD	5.73/5.37/3.36	3.24/2.91/2.53	2.93/3.35/1.45	8.74/1.43/7.87
t-value/p-value	7.00/<0.001	6.56/<0.001	4.80/<0.001	18.53/<0.001
Effect size	d = 1.29 (95%CI: 0.27–2.30)	d = 1.64 (95%CI: 0.57–2.71)	d = 0.67 (95%CI: -0.26–1.61)	d = 7.09 (95%CI: 4.45–9.72)

## Discussion

In this report, using the SAMR and interview methods, an intervention process similar to that used in previous studies [11] was conducted, and the state of achievement motivation as well as the characteristics of behavioral change were examined through a case series of 11 participants. All participants in this report were women who had received the same diagnosis and surgical procedure and underwent occupational therapy interventions by the co-author. As a result of the intervention, large or medium effects were observed in the total SAMR scores and each factor score. Because achievement motivation has been reported to influence not only health-related quality of life but also participation in personally meaningful daily activities [18], a process was implemented through interviews to set goals connected to such activities (Figure 1, step 2), which led to goal setting at the activity and participation level rather than merely at the level of body functions. This interview method, as in previous studies [11], is considered to have contributed to improvements in achievement motivation and behavioral change. Furthermore, compiling the collaboratively agreed-upon daily goals, action plans, and the support available from those around the participants into a booklet helped to visualize aspects such as metacognition and behavioral regulation (Figure 1, step 3), which are important for self-regulated learning [19]. In other words, it is thought that, by following a process where the participants could adjust their own actions, reflect on themselves, and seek appropriate support from people close to them to solve problems, the intervention led to support that enabled them to actively engage on their own initiative.

In the correlation analysis between the change in FIM scores and the initial SAMR total score in this report, statistical significance was not observed. However, the effect size was found to be a medium effect. Therefore, by appropriately setting the sample size and verifying the intervention effect, it is possible that the degree of improvement in activities of daily living may vary depending on the level of achievement motivation at the initial stage. In this report, it is considered that participants with high achievement motivation at the initial evaluation tended to look ahead to life at home after discharge, adhere to goals and action plans, and prefer to engage in activities with their own creative ideas. On the other hand, participants with moderate achievement motivation at the initial evaluation often made abstract complaints, and there is a possibility that they may have difficulty focusing on their future lives. They may also be more likely to have a passive mindset, expecting the occupational therapists to make things better or to provide treatment. Therefore, occupational therapists need to first guide the patients to focus on their daily lives, activities, and social participation when setting goals. It also makes it necessary to assist by asking questions that help them set specific goals. Furthermore, throughout the intervention process, it is important to expect participants to become able to think about their own goals, gradually shift to more open-ended questions, and appropriately use confirmation and prompting interventions so that participants can learn how to set their own goals and action plans.

Previous studies targeting elderly residents in the community have reported that, among the various factors influencing achievement motivation, there are no gender differences, and the factors with the strongest impact are personality traits (extraversion, conscientiousness, and openness) [9]. Studies targeting the general adult population have also clarified that extraversion, conscientiousness, and openness are positively associated with achievement motivation [20,21]. Furthermore, factors such as higher frequency of going out and living alone have also been suggested to increase achievement motivation [9]. Therefore, it is necessary to consider basic information such as the participant’s innate characteristics and family structure, as well as their lifestyle before the onset of illness or hospitalization, as these may affect their motivation to engage in rehabilitation. Even taking these influences into consideration, further research is needed to determine whether the kind of intervention process described in this report can positively impact motivation and behavioral modification.

The strength of this study lies in its presentation of a systematic approach that combines SAMR with semi-structured interviews to enhance motivation and participation among patients after THA. This approach aims to foster individual autonomy and improve ADL and could serve as a meaningful method for understanding and supporting individuals’ psychological and motivational states beyond the specific condition targeted in this study. These findings suggest that this intervention can effectively promote individuals’ intrinsic motivation and contribute to better rehabilitation outcomes. By empowering patients to actively participate in their rehabilitation process, this approach is considered to be well-aligned with the principles of person-centered therapy.

In addition, this report has several limitations. First, the results were obtained at a single hospital by the occupational therapist, who is a co-author, and all participants were female, so there is a significant possibility of selection bias. Furthermore, we were unable to conduct detailed comparisons regarding the surgical procedures performed, the types of artificial joints used, the size of the hospital, the structure and ratio of therapists, or background information such as the region or family circumstances of the patients. Moreover, there was no strict standardization regarding how the interview content or therapists’ strategies were adapted based on the SAMR scores, or in terms of self-assessment, the time and frequency required for the interviews, or the duration of the intervention. As a limitation of the study design, it should be noted that this study explored the effects of the intervention, and therefore, any effects indicated by this intervention are only suggestive.

Taking these strengths and limitations into account, we aim to further develop this research by examining the minimal clinically important difference [22] for changes in the SAMR score, designing sample sizes based on the effect sizes reported in this study, and conducting empirical studies to verify the intervention effects through comparisons with control groups. Additionally, it is necessary to carefully select the information to be collected and standardize the hospitals and therapists involved. Since semi-structured interviews heavily depend on the interpersonal skills and interpretations of the therapists, it is also essential to establish a standardized training system for interviewing methods.

## Conclusions

This case series demonstrated that the use of the SAMR combined with a semi-structured interview method contributed to improvements in achievement motivation and daily activity performance among patients after THA. The intervention process enabled participants to set personal, meaningful goals and action plans, thereby fostering self-regulation and behavioral modification. These findings highlight the potential role of occupational therapists in supporting patient-centered rehabilitation through indirect approaches that enhance motivation. However, further studies with larger and more diverse samples are needed to confirm these preliminary findings.
